# Clinical Characteristics, Management, and Outcomes of Colitis-Associated Colorectal Cancer and the Comparison With Sporadic Colorectal Cancer in Taiwan

**DOI:** 10.14309/ctg.0000000000000798

**Published:** 2024-12-05

**Authors:** Hsin-Yun Wu, Meng-Tzu Weng, Jen-Wei Chou, Hsu-Heng Yen, Chun-Chi Lin, Feng-Fan Chiang, Chen-Shuan Chung, Wei-Chen Lin, Chen-Wang Chang, Puo-Hsien Le, Chia-Jung Kuo, Ching-Pin Lin, Wen-Hung Hsu, Chiao-Hsiung Chuang, Tzung-Jiun Tsai, I-Che Feng, Shu-Chen Wei, Tien-Yu Huang

**Affiliations:** 1 Division of Gastroenterology and Hepatology, Department of Internal Medicine, National Taiwan University Hospital Jinshan Branch, New Taipei City, Taiwan;; 2 Division of Gastroenterology and Hepatology, Department of Internal Medicine, National Taiwan University Hospital, Taipei, Taiwan;; 3 Department of Medical Research, National Taiwan University Hospital Hsin-Chu Branch, HsinChu, Taiwan;; 4 Center for Digestive Medicine, Department of Internal Medicine, China Medical University Hospital, Taichung, Taiwan;; 5 Division of Gastroenterology, Department of Internal Medicine, Changhua Christian Hospital, Changhua, Taiwan;; 6 Division of Colon and Rectal Surgery, Department of Surgery, Taipei Veterans General Hospital, Taipei, Taiwan;; 7 Division of Colorectal Surgery, Department of Surgery, Taichung Veterans General Hospital, Taichung, Taiwan;; 8 Division of Gastroenterology and Hepatology, Department of Internal Medicine, Far Eastern Memorial Hospital, New Taipei City, Taiwan;; 9 Division of Gastroenterology, Department of Internal Medicine, Mackay Memorial Hospital, Taipei, Taiwan;; 10 Department of Gastroenterology and Hepatology, Chang Gung Memorial Hospital, Linkou Branch, Taoyuan, Taiwan;; 11 Division of Gastroenterology and Hepatology, Department of Internal Medicine, Chung Shan Medical University Hospital, Taichung, Taiwan;; 12 Division of Gastroenterology, Department of Internal Medicine, Kaohsiung Medical University Chung-Ho Memorial Hospital, Kaohsiung, Taiwan;; 13 Department of Internal Medicine, National Cheng Kung University Hospital, National Cheng Kung University College of Medicine, Tainan, Taiwan;; 14 Division of Gastroenterology and Hepatology, Department of Internal Medicine, Kaohsiung Veterans General Hospital, Kaohsiung, Taiwan;; 15 Division of Gastroenterology and Hepatology, Department of Internal Medicine, Chi-Mei Medical Center, Tainan, Taiwan;; 16 Division of Gastroenterology, Department of Internal Medicine, Tri-Service General Hospital, National Defense Medical Center, Taipei, Taiwan.

**Keywords:** inflammatory bowel disease, colitis-associated colorectal cancer, sporadic colorectal cancer, surveillance, outcomes

## Abstract

**INTRODUCTION:**

We explored the clinical characteristics, treatment, and outcomes of colitis-associated colorectal cancer (CAC) and compared with sporadic colorectal cancer in Taiwan.

**METHODS:**

In this retrospective study spanning 1987–2022, CACs diagnosed according to endoscopic and pathological reports from 14 tertiary centers were reported to our cohort. Clinical demographics, endoscopic findings, histological results, treatment modalities, and outcomes were analyzed. Sporadic colorectal cancer data were retrieved from the Cancer Registry Annual Report, Ministry of Health and Welfare, Taiwan.

**RESULTS:**

We enrolled 65 patients with CAC (median age: 56 years; male: 66.2%). Distal colon was the most common tumor location (41.5%). Of patients with ulcerative colitis, 77.2% had extensive colitis, and 76.5% had Mayo endoscopic subscores of ≥2. Moreover, 50% of lesions were nonpolypoid with indistinct borders in 66.7%. Signet-ring cell subtype consisted of 12.3%. Surveillance colonoscopy adherence was 78.4%, yet 51.3% interval cancers occurred. Disease stage 0–4 distribution was 15%, 20%, 13.3%, 20%, and 31.7%, respectively. Endoscopic resection was feasible for 14%, whereas 67.7% required surgery. During follow-up (median: 21.5 months), we recorded 23.2% recurrence and 34.5% mortality. Lesions with indistinct borders were associated with adverse outcomes (adjusted odds ratio = 11.5 [1.35–98.16]). Colitis-associated rectal cancers, diagnosed later (*P* < 0.001), had worse outcomes than sporadic rectal cancers.

**DISCUSSION:**

This is the largest Asian CAC cohort study, emphasizing the need for stringent disease control, improving detection, and reducing interval cancers. Signet-ring cell subtype was prevalent. Rectal colitis-associated cancers were diagnosed later with poorer outcomes than sporadic rectal cancers.

## Graphical abstract

**Figure:**
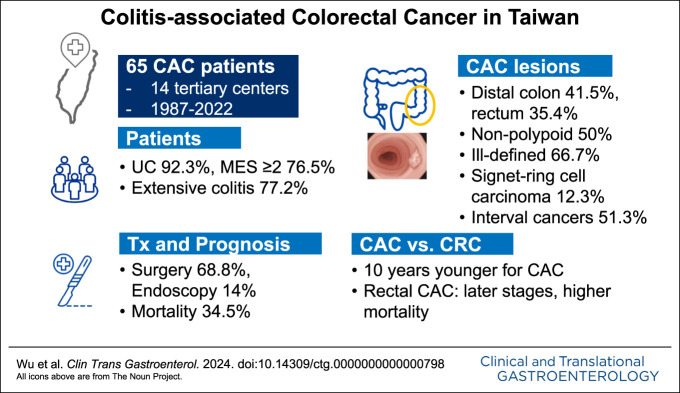

## INTRODUCTION

Patients with inflammatory bowel disease (IBD) have higher risks of colitis-associated colorectal cancer (CAC). The landmark publication by Eaden et al (1) in 2001 reported CAC prevalence of 3.7% in ulcerative colitis (UC) and 5.4% in extensive colitis. The cumulative risks increased with disease duration. Advances in dysplasia recognition, inflammation control, and surveillance have, however, led to a decreased incidence of CAC (2,3). A 2013 meta-analysis by Lutgens et al (3) revealed that pooled cumulative risks of CAC for UC and Crohn's disease (CD) were 0.7% at 10 years, 2.6% at 20 years, and 6.6% after 20 years. Nonetheless, CAC remains a devastating complication, resulting in worse overall and cancer-specific survival compared with sporadic colorectal cancer (CRC) (4).

The incidence and prevalence of IBD in Asia are increasing. A meta-analysis by Bopanna et al (5) disclosed cumulative CAC risk of 0.02% at 10 years, 4.81% at 20 years, and 13.91% at 30 years in Asian UC populations. Unlike Western trends, there was no declining trend in CAC risk over time. Possible explanations are limited effective treatments and well-established surveillance programs in Asia.

Taiwan experiences a similar trend in rising IBD incidence (6). In 2012, Wei et al (7) determined the incidence of CAC was 1.5% among patients with UC at a tertiary center, with cumulative risk of 0% at 10 years, 6% at 20 years, and 12.3% at 30 years, consistent with the meta-analysis of Asian populations. By contrast, Kuo et al (8) conducted a population-based study in 2015, reporting a much lower incidence of CAC; specifically, 0.24% for UC and 0.19% for CD. Population-based studies lack in-depth clinical analyses, and single-center studies may lack generalizability. In addition, CAC studies from Asia have few case numbers. Accordingly, we conducted a nationwide multicenter study to identify the clinical features, management, and outcomes of CAC in Taiwan.

Contrary to the classic adenoma carcinoma sequence, the major molecular mutation of CAC appeared in different timing and frequency. For example, loss of *P53* function was acquired rather earlier, whereas *APC* gene mutation occurred later in the process (9). Through transcriptomic analysis, CRCs could be classified into 4 consensus molecular subtypes (CMS). CMS1 group, also called microsatellite instability immune subtype, accounted for similar percentages in sporadic CRC and CAC. Although CRC comprises more of the epithelial cell differentiation CMS2, CAC shifted toward the mesenchymal differentiation CMS4 (10). These differences may influence the tumor locations, manifestations, and the “nonconventional dysplasias” in histology, such as hypermucinous tumors (11). In the current study, we also aim to compare the clinical characteristics and stages between the CAC and sporadic CRC.

## MATERIALS AND METHODS

### Study design and definition

This retrospective cohort study adhered to the Declaration of Helsinki and obtained approval from ethical committees. The informed consent was waived because of the retrospective design.

CAC was diagnosed according to endoscopic and pathological reports, encompassing high-grade dysplasia because of its progression to cancer if untreated. Disease staging followed the *American Joint Committee on Cancer 8th Edition* guidelines (12). The early disease stage was defined as stage 2 and earlier, whereas the late disease stage was defined as stages 3 and 4. Local gastroenterologists or colorectal surgeons supplied information on demographics, endoscopic and histological results at the time of diagnosis, history of IBD-related medications, treatment modalities, and outcomes. The physicians involved were specialists in IBD and endoscopy in the tertiary centers. For UC, disease extent was classified into proctitis, left-sided colitis, and extensive colitis; for CD, disease extent was determined using the Montreal classification system. Disease duration was defined as the interval between the diagnosis of CAC and the first reported onset of IBD-related symptoms. Descriptions on lesion morphology (polypoid or nonpolypoid) and border (distinct or indistinct) aligned with the Surveillance for Colorectal Endoscopic Neoplasia Detection and Management in Inflammatory Bowel Disease Patients: International Consensus Recommendations (SCENIC) (13). Adherence to surveillance referred to patients receiving colonoscopies within the recommended intervals based on their individual risk stratification. World Endoscopy Organization defined interval cancer as those identified before the next recommended surveillance examination (14). Surveillance guideline was first introduced in 2002 by Eaden et al (15), which evolved over the years. According to the consensus guideline established by our Taiwan Society of IBD, surveillance for CAC in individuals with extensive-type UC should commence 8 years after diagnosis, whereas those with left-sided UC should initiate 12 years after diagnosis, without stratifying the different interval for different risk categories (16). Therefore, for our assessment, we selected the original guideline from 2002 and also American Gastroenterological Association recommendation from 2010 to 2021 (17,18). We then evaluated our patient's adherence to surveillance protocol and examined the interval cancer rates among those who adhered to the guidelines applicable at the moment of their diagnosis. Surgical procedures are categorized as colectomy (including all colectomy methods preserving the rectum), proctocolectomy (including abdominal perineal resection), and palliative ostomy. Recurrence and mortality were recorded. Prognosis was classified as either good or poor (involving disease progression, recurrence, or mortality). Data authentication involved 3 authors (H.Y.W., S.C.W., and T.Y.H.). Information on sporadic CRC was retrieved from the Taiwan's Ministry of Health and Welfare Cancer Registry Annual Report (19).

### Statistical analysis

Continuous variables are presented as medians (ranges) and categorical variables as numbers (percentages). Differences between groups were evaluated using the Mann-Whitney *U* test or χ^2^ test where appropriate. Clinically relevant and significant variables associated with poor prognosis in univariate analysis were included in multivariable logistic regression analysis. Odds ratios (ORs) and 95% CIs were calculated. All statistical analyses were performed using IBM SPSS for Windows (version 25.0; IBM, Armonk, NY). All tests were 2-sided, and a *P* value of <0.05 was considered statistically significant.

## RESULTS

### Patient demographic characteristics

A total of 65 patients with CAC, predominantly male (66.2%) with a median age of 56 years, were included between 1987 and 2022 (Table 1). The majority (n = 60; 92.3%) of the patients had UC. The risk of CAC increased with the duration of IBD, with a median duration of 13 years (see Figure, Supplementary Digital Content 1, http://links.lww.com/CTG/B232). Among patients with UC, 77.2% had extensive colitis, and 76.5% had Mayo endoscopic subscores ≥2 at the time of CAC diagnoses. All 5 patients with CD were ileocolonic type, whereas one had upper gastrointestinal tract involvement. Three patients with CD were older than 40 years (A3), and 2 were aged between 17 and 40 years (A2). Phenotypically, 3 presented as penetrating (B3), one as stricturing (B2), and one as nonstricturing and nonpenetrating (B1). The patient with B2 presentation also had perianal disease.

**Table 1.** Clinical demographics of enrolled patients with CAC (N = 65)

Parameters	Numbers or median
Age, yr	56 (22–84)
Male	43 (66.2%)
IBD subtype, UC	60 (92.3%)
IBD to CAC interval, yr	13 (0–39)
UC extent (n = 57)	
Proctitis	2 (3.5%)
Left-sided colitis	11 (19.3%)
Extensive colitis	44 (77.2%)
Mayo endoscopic subscore (n = 51)	
0	6 (11.8%)
1	6 (11.8%)
2	18 (35.3%)
3	21 (41.2%)
Montreal classification, age (n = 5)	
A2	2 (40%)
A3	3 (60%)
Montreal classification, location (n = 5)	
L3	5 (100%)
+L4	1 (20%)
Montreal classification, behavior (n = 5)	
B1	1 (20%)
B2/B2P	1 (20%)
B3	3 (60%)
CRC family history (n = 62)	3 (4.8%)
Primary sclerosing cholangitis (n = 62)	1 (1.6%)
Pseudopolyps (n = 62)	28 (45.2%)
Medication (n = 64)	
Mesalazine	59 (92.2%)
Azathioprine	24 (37.5%)
Steroid	38 (59.4%)
Biologics	14 (21.9%)
Elevated CEA (n = 57)	24 (42.1%)
CEA, ng/mL	4.09 (0.6–216)
Colonoscopy interval, mo	12 (0–141)
Adherence to surveillance (n = 51)^a^	40 (78.4%)

Data are expressed as numbers (percentages) or medians (ranges).

CAC, colitis-associated colorectal cancer; CEA, carcinoembryonic antigen; CRC, colorectal cancer; IBD, inflammatory bowel disease; UC, ulcerative colitis.

a Adherence to surveillance referred to the proportion of patients who received colonoscopies during the recommended interval based on their individual risk stratification, in accordance with the guidelines applicable at the moment of their diagnosis.

Established risk factors such as a family history of CRC, primary sclerosing cholangitis, and pseudopolyps were noted in 4.8%, 1.6%, and 45.2% of the patients, respectively. Medicationwise, 92.2% received mesalazine; 59.4% had previous steroid exposure, and 21.9% received biological agents. The median carcinoembryonic antigen level was 4.09 ng/mL, which was higher than the reference range in 42.1% of the patients. We assessed our patients' adherence if their collected information allowed adequate risk stratification. We found that 40 patients (78.4%) received surveillance colonoscopy within the recommended interval and the median colonoscopy interval was 12 months.

### Characteristics and management of CAC

Regarding CAC locations, the left colon (distal to the splenic flexure) was the most frequent location (41.5%), followed by the rectum (35.4%) and the right colon (23.1%) (Table 2). Nonpolypoid lesions accounted for 50%, and 66.7% had indistinct borders. White-light endoscopy detected 85.9% of the lesions. Chromoendoscopy was not routinely performed, and when performed, it was often to identify lesion characteristics and define the margins. Lesions required virtual or dye-based chromoendoscopy for better visualization accounted for 4.7% each. In Supplementary Figure 2A (see Figure, Supplementary Digital Content 2, http://links.lww.com/CTG/B232), a pseudodepressed nongranular type lateral spreading tumor was illustrated as an example of a nonpolypoid lesion with a distinct border. In addition, another nonpolypoid lesion was observed under white-light endoscopy, and its initially indistinct border became clearly visible after virtual chromoendoscopy with narrow-band imaging, as depicted in Supplementary Figures 2B and 2C, (see Supplementary Digital Content, http://links.lww.com/CTG/B232). The remaining 4.7% of the lesions were diagnosed using methods other than endoscopy (1 received surgery because of colon perforation, 1 underwent surgical dilatation because of anal stenosis, and 1 through compatible image and cell block from paracentesis). No synchronous CAC was observed. Among the CAC, 48.3% were early stage, and 51.7% were late-stage. The most common histological type was adenocarcinoma (86.2%). Signet-ring cell carcinoma and mucinous adenocarcinoma accounted for 12.3% and 9.2%, respectively. High-grade dysplasia accounted for 9.2%. Poorly differentiated carcinoma and adenosquamous cell carcinoma were less common histological types.

**Table 2.** Clinical characteristics, management, and outcomes of CAC (N = 65)

Parameters	Numbers or median
CAC site	
Colon, proximal to splenic flexure	15 (23.1%)
Colon, distal to splenic flexure	27 (41.5%)
Rectum	23 (35.4%)
Endoscopy	
Modality, white-light detection (n = 64)	55 (85.9%)
Nonpolypoid (n = 54)	27 (50%)
Indistinct border (n = 54)	36 (66.7%)
Cancer stage, AJCC 8th (n = 60)	
Stage 0	9 (15%)
Stage 1	12 (20%)
Stage 2	8 (13.3%)
Stage 3	12 (20%)
Stage 4	19 (31.7%)
Pathology	
Adenocarcinoma^a^	56 (86.2%)
Signet-ring cell carcinoma^a^	8 (12.3%)
Mucinous adenocarcinoma^a^	6 (9.2%)
High-grade dysplasia	6 (9.2%)
Poorly differentiated carcinoma	2 (3.1%)
Adenosquamous carcinoma	1 (1.5%)
Management (n = 64)	
Endoscopic treatment	9 (14%)
EMR	2 (3.1%)
ESD	2 (3.1%)
Polypectomy	5 (7.8%)
Surgical treatment	44 (68.8%)
Colectomy^b^	38 (59.4%)
Proctocolectomy^b^	4 (6.3%)
Palliative ostomy	2 (3.1%)
Chemotherapy	29 (45.3%)
Radiotherapy	15 (23.4%)
Interval cancer^c^ (n = 39)	20 (51.3%)
Follow-up time, mo (n = 58)	21.5 (1–420)
Recurrence (n = 56)	13 (23.2%)
Local	3 (5.4%)
Distant	10 (17.9%)
Mortality (n = 58)	20 (34.5%)
Mortality time, mo (n = 18)	14.5 (2–49)
Disease-free time, mo (n = 55)	13 (0–420)

Data are the pooled results obtained for patients with UC and CD and are expressed as numbers (percentages) or medians (ranges).

AJCC, American Joint Committee on Cancer; CAC, colitis-associated colorectal cancer; CD, Crohn's disease; EMR, endoscopic mucosal resection; ESD, endoscopic submucosal dissection; UC, ulcerative colitis.

a Signet-ring cell carcinoma and mucinous adenocarcinoma were included as histological subtypes of adenocarcinoma. Owing to their aggressive nature, they were singled out for comparison.

b Curative surgical procedures were classified as colectomy procedures if the rectum was preserved or proctocolectomy procedures if the rectum was resected.

c Interval cancer was defined as those identified before the next recommended surveillance examination (14). Interval cancer rates were examined among patients who adhered to surveillance guidelines.

Because of indistinct borders and late-stage presentation, endoscopic resections were only eligible for 14% of the patients (polypectomy: 7.8%, endoscopic mucosal resection: 3.1%, and endoscopic submucosal dissection: 3.1%). Surgeries were performed in 68.8% of patients, including 4.5% for palliative intent. Even for high-grade dysplasia, one patient received colectomy because of ill-defined borders. In addition, 45.3% received chemotherapy, and 23.4% underwent radiotherapy.

Of those adhering to surveillance, 20 (51.3%) were later diagnosed as having interval cancers. Notably, steroid use significantly correlated with interval cancer development in univariate analysis (*P* = 0.03).

### Outcomes and prognostic factors for CAC

**Figure 1.**
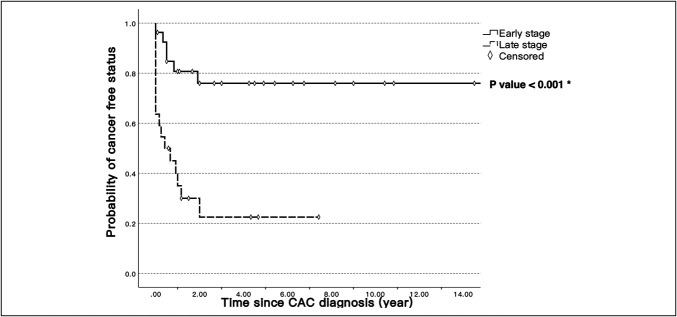
Probability of colitis-associated colorectal cancer-free status after diagnosis. Cancer-free status was defined as not having disease progression, recurrence, or mortality. The Kaplan-Meier curves were distinctly different between early and late stages (*P* < 0.001). The asterisk (*) denotes statistical significance.

During a median 21.5-month follow-up, we recorded a 23.2% recurrence rate and 34.5% mortality. Among the patients with recurrence, 76.9% had distant recurrence, most commonly peritoneal carcinomatosis (n = 6). A Kaplan-Meier curve (Figure 1) plotted CAC-free probability, defined as not having disease progression, recurrence, or mortality. At early disease stages, the curve exhibited a gentle slope and plateaued in the second year after diagnosis. By contrast, at late disease stages, the curve initially sloped downward, representing the large proportion of patients who never achieved disease-free status (*P* < 0.001). Median CAC-free duration was 13 months, and median time to death was 14.5 months. Among 29 early stage patients, 23 patients (79.3%) attained disease-free status, with median duration increasing to 34 months. Median time to death increased to 23 months.

Table 3 outlined significant poor prognosis risk factors identified through univariate analysis. In the poor-prognosis group, tumor-related factors including lesions with indistinct borders (87.5%), rectal location (57.7), late-stage diagnosis (76%), and presence of signet-ring cell components (26.9%) were significantly more common than in the good-prognosis group. Regarding treatment-related risk factors, patients unsuitable for endoscopic treatment (*P* = 0.03) and required chemotherapy (*P* = 0.001) were more likely to develop poor prognoses. Multivariable logistic regression highlighted lesions with indistinct borders were 11.5 times more likely to lead to adverse outcomes (adjusted odds ratio [OR] = 11.52; 95% confidence interval [CI]: 1.35–98.16; *P* = 0.03).

**Table 3.** Prognostic factors for CAC

	Good prognosis^a^	Poor prognosis^a^	Univariate analysis	Multivariable logistic regression	
	(N = 32)	(N = 26)	*P* value	Adjusted OR (95% CI)	*P* value
Indistinct border	13 (N = 26, 50%)	21 (N = 24, 87.5%)	0.005*	11.52 (1.35–98.16)	0.03*
CAC site, rectum	6 (18.8%)	15 (57.7%)	0.002*	5.49 (0.85–35.33)	0.07
CAC stage			<0.001*		
Early (0–2)	23 (N = 31, 74.2%)	6 (N = 25, 24%)			
Late (3–4)	8 (N = 31, 25.8%)	19 (N = 25, 76%)		1.25 (0.16–9.76)	0.83
Pathology			0.009*		
Adenocarcinoma^b^	25 (78.1%)	17 (65.4%)			
SRC^b^	1 (3.1%)	7 (26.9%)		2.09 (0.14–30.77)	0.59
HGD	6 (18.8%)	0 (0%)			
Others^c^	0 (0%)	2 (7.6%)			
Endoscopic treatment	8 (25%)	1 (3.8%)	0.03*	1.40 (0.09–22.08)	0.81
Chemotherapy	9 (28.1%)	19 (73.1%)	0.001*	5.28 (0.62–44.95)	0.13

Data are expressed as numbers (percentages). Only the statistically significant clinical parameters in the univariate analysis are listed. The asterisk (*) denotes statistical significance.

CAC, colitis-associated colorectal cancer; CI, confidence interval; HGD, high-grade dysplasia; OR, odds ratio; SRC, signet-ring cell carcinoma.

a A poor-prognosis group comprised patients whose cancer progressed, recurred, or led to mortality, whereas the good-prognosis group comprised those without such outcomes.

b Signet-ring cell carcinoma was singled out from the adenocarcinoma subtypes for comparison owing to its aggressive nature.

c Other histological types included adenosquamous cell carcinoma and poorly differentiated carcinoma.

### Comparison between CAC and sporadic CRC

The national CRC screening program of Taiwan started in 2004, providing biennial fecal immunochemical tests universally to people aged 50–69 years. People who tested positive would be invited for colonoscopy. The upper age limit was extended to 75 years in 2014. The Ministry of Health and Welfare of Taiwan provided cancer registry data since 1995, with updates through 2020. The data set has undergone progressive augmentation, incorporating the incidence rates across different cancer stages since 2008. We limited our analysis to patients diagnosed between 2008 and 2020 within our cohort for a direct comparison with sporadic CRC population during the same period (Table 4) (19). Importantly, this approach avoided biases from the evolving landscape of surveillance, diagnosis, and treatment modalities.

**Table 4.** Comparison between CAC and sporadic CRC

	Colon		Rectum	
	CAC	CRC^a^	CAC	CRC^a^
Number	28 (66.7%)	123,968 (62.6%)	14 (33.3%)	73,932 (37.4%)
Age, yr	57.5	67	52	64
Male	19 (67.9%)	66,661 (53.8%)	13 (92.9%)	45,285 (61.3%)
Pathology				
Adenocarcinoma	25 (89.3%)	116,083 (93.6%)	10 (71.4%)	66,946 (90.6%)
SRC^b^	2 (7.1%)	NA	4 (28.6%)	NA
Cancer stage		*P* = 0.03*		*P* < 0.001*
Stage 0	4 (14.8%)	28,322 (22.8%)	1 (7.1%)	9,438 (13.8%)
Stage 1	8 (29.6%)	17,924 (14.5%)	2 (14.3%)	14,570 (19.7%)
Stage 2	6 (22.2%)	24,840 (20.0%)	0 (0%)	11,271 (15.2%)
Stage 3	3 (11.1%)	28,627 (23.1%)	3 (21.4%)	21,285 (28.8%)
Stage 4	6 (22.2%)	24,255 (19.6%)	8 (57.1%)	12,401 (16.8%)
Mortality	7 (26.9%)	51,006 (41.1%)	8 (57.1%)	19,777 (26.8%)

The asterisk (*) denotes statistical significance. CAC, colitis-associated colorectal cancer; CRC, colorectal cancer; SRC, signet-ring cell carcinoma.

a CRC data were retrieved from the Cancer Registry Annual Report provided by the Ministry of Health and Welfare of Taiwan (19).

b Signet-ring cell carcinoma was singled out from adenocarcinoma subtypes for comparison owing to its aggressive nature.

**Figure 2.**
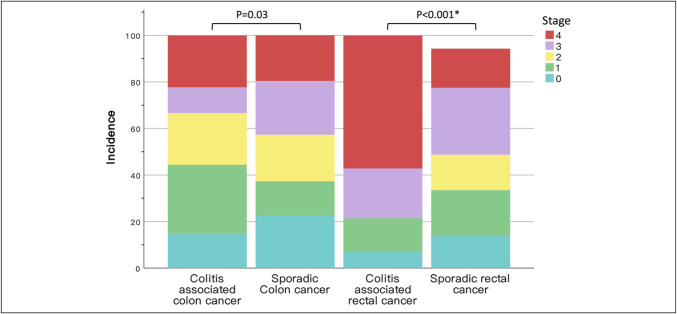
Distribution of colitis-associated and sporadic colorectal cancer stages. The incidence of CAC was stratified from stages 0–4 and subsequently compared with the incidence of colon and rectal cancer among the CRC population. The stage distributions were significantly different for rectal cancers (*P* < 0.001). CAC, colitis-associated colorectal cancer; CRC, colorectal cancer. The asterisk (*) denotes statistical significance.

Stratifying patients by colonic or rectal location revealed 62.6% of sporadic CRC as colon cancers and 37.4% as rectal cancers. Similarly, in our CAC cohort, 33.3% had rectal cancers. Cancer onset occurred approximately a decade earlier in the CAC cohort for both colon and rectal cancers compared with the sporadic CRC population. Male predominance was consistent in both CAC and sporadic CRC, regardless of cancer location. Adenocarcinoma constituted 93.6% of sporadic colon cancers and 90.6% of rectal cancers, compared with 89.3% of colon CAC cases and 71.4% of rectal CAC cases in our cohort. Notably, 7.1% of the colon CAC subtypes and 28.6% of the rectal CAC subtypes exhibited signet-ring cell components. However, information regarding signet-ring cells was unavailable in the cancer registry. Interestingly, a notable disparity in disease stage distribution was observed between CAC and sporadic CRC, particularly with a higher prevalence of early stage disease in colon CAC (*P* = 0.03) and later stages in rectal CAC (*P* < 0.001; Figure 2). These divergences in stage distribution directly reflected on the mortality rates. Specifically, the mortality rates for colonic CAC and sporadic cancers were 26.9% and 41.1%, respectively, whereas those for rectal CAC and sporadic cancers were 57.1% and 26.8%, respectively.

## DISCUSSION

This study analyzed data from 65 patients with CAC, rendering it the largest Asian CAC cohort to date. This was an especially valuable cohort considering the low IBD prevalence in Taiwan. The most updated epidemiology report in 2015 revealed prevalence of UC and CD as 12.8 and 3.9/100,000, respectively (6). We provided detailed information regarding the morphology of lesions, CAC management, and long-term follow-up outcomes. Moreover, we identified several key clinical features of CAC. First, patients with UC with extensive colitis and severe inflammation were at a high risk of CAC. Second, vigilance during endoscopy was crucial for detecting nonpolypoid lesions with indistinct borders. Furthermore, CAC predominantly occurred in the left colon and rectum. Surgery was the prevailing treatment. In addition, we should be aware of interval cancers. Compared with the sporadic CRC in Taiwan, CAC was diagnosed younger, more commonly exhibiting signet-ring cell subtype, and worse outcomes for rectal CAC.

We compared our study with retrospective cohort studies from India, China, Korea, and Japan (Table 5) (20–24). The median age at CAC onset aligned with China's study, but was higher than others. Male predominance was universal. CAC most often took the form of extensive colitis in UC and an ileocolonic type in CD. Our patients had a shorter duration of CAC development than did those in the other studies. Notably, 24.6% of our patients developed CAC within 8 years of the onset of IBD symptoms. Hata et al (25) also reported that 17% of their patients developed CAC within this timeframe. Although the prevalence of IBD is increasing in Asia, it is still lower than that in Western countries. Lack of public awareness of the symptoms of IBD may delay medical attention. Our study involved referral centers; hence, delays from patients and primary physicians may contribute to the prolongation of the unrecognized and untreated inflammation periods and the delay of timely surveillance colonoscopies.

**Table 5.** Asian colitis-associated colorectal cancer studies

Author	Year	Site	IBD subtype	Patient number	CAC number	Age, yr^a^	Male, %	Extensive disease^b^ %	IBD CAC interval, yr^a^	CAC risk, %		
										10 yr	20 yr	30 yr
Bopanna et al (20)	2004–2015	India, multicenter	UC	1,012	20 (2%)	35	70	80	18.7	1.5	7.2	23.6
Gong et al (21)	1998–2009	China, multicenter	UC	3,922	34 (0.9%)	57.5	54	64.7	12.8	1.2	3.6	14.4
Lee et al (22)	1989–2013	Korea, single center	UC	2,798	18 (0.6%)	48	53.7	40.9	15	0.3	3.4	9.4
			CD	2,414	12 (0.5%)	32	72.3	72.5	12.5	0.3	3.8	3.8
Yoshino et al (23)	2003–2013	Japan, multicenter	UC	2,137	43 (2%)	53	69.8	79.1	13	NA	NA	NA
Wu et al	1987–2022	Taiwan, multicenter	UC	4,674^c^	60 (1.3%)	56.5	66.7	77.2	13	NA	NA	NA
			CD	2,080^c^	5 (0.2%)	54	60	100	10	NA	NA	NA

CAC, colitis-associated colorectal cancer; CD, Crohn's disease; IBD, inflammatory bowel disease; NA, not available; UC, ulcerative colitis.

a Age and IBD-to-CAC intervals are expressed as medians, except for the mean value reported by Bopanna et al.

b Extensive disease refers to extensive colitis in UC and ileocolonic CD.

c The number of patients was retrieved from the catastrophic illness certification registry provided by the Ministry of Health and Welfare of Taiwan (24). The prevalence of CAC was then estimated as 1.3% for UC and 0.2% for CD.

Most of our patients with UC experienced extensive colitis and active inflammation. Inflammation was not well controlled because 59.4% of our patients were previously exposed to steroids. Oxidative stress from chronic inflammation causing DNA damage is the generally perceived carcinogenesis of CAC (26). Previous studies revealed that extensive disease (27) and active inflammation (28) were risk factors for CAC. Therefore, improving inflammatory control strategies is imperative. The introduction of biological agents in our National Health Insurance in 2011 may impact this but warrants longer-term follow-up for evaluation.

The distinct molecular pathogenesis of CAC gives rise to its distinct morphological and histological presentations. This study found that 50% of CAC lesions were nonpolypoid, and 66.7% had indistinct borders. Mutaguchi et al (29) demonstrated differences in endoscopic findings of CAC and sporadic neoplasia (*P* < 0.001). They reported that only 25.8% of the CAC lesions in their study were polypoid and that 62.1% of the lesions had indistinct margins. According to international consensus in 2015, chromoendoscopy should be performed over standard-definition white-light endoscopy for lesion screening (13). However, a recent meta-analysis demonstrated that high-definition white-light endoscopy is equally effective (30). The most recent European Crohn's and Colitis Organisation guideline also recommended either chromoendoscopy or high-definition white-light endoscopy (31). In our analysis, 12.3% were diagnosed with signet-ring cell carcinoma, known for poor outcomes and a low rate of R0 resections (4). The prevalence of signet-ring cell carcinoma has been reported to be approximately 0.16% in a tertiary center in Taiwan (32) and 0.6% in an American Veteran database (33).

Surveillance colonoscopy significantly reduces CAC development and associated mortality by enabling early detection (34). In our cohort, 78.4% underwent surveillance within the recommended interval, with nonadherence possibly attributed to bowel preparation inconvenience, procedural risk concerns, or financial considerations. Physician unawareness could also contribute because adherence was 35.7% in referred cases compared with 75.6% in cases followed up at the centers. Among adherents, 51.3% met the interval cancer definition, contrasting with an 8.2% postcolonoscopy CRC prevalence in a meta-analysis (35). Furthermore, among patients with interval cancer, 10 (50%) had late-stage disease. Colonoscopy surveillance programs for CAC in the United Kingdom and the Netherlands have also demonstrated high percentages of interval cancers (53% and 45%, respectively) (36,37). Missed lesion was the most common etiology in the Dutch cohort. Insufficient data on physician adherence to the recommendations of endoscopic modalities, quality benchmarks (cecal intubation, bowel preparation scores, etc), and previous endoscopic resection details hindered the comprehensive evaluation of the causes for interval cancers. Steroid use, indicating ongoing severe inflammation, was a significant risk factor for interval cancer (*P* = 0.03). Emphasizing adherence to high-definition white-light endoscopy or chromoendoscopy and surveillance examination quality is crucial because of the heightened risk of interval cancers. In the future, artificial intelligence tools (38) may help reduce missed lesions and interval cancers.

Advancements in endoscopy have improved optical diagnosis methods and provided feasible therapeutic options, such as endoscopic submucosal dissection for CAC (39). A Japanese study (23) reported that 57.1% of patients received a diagnosis at stage 1 or earlier, and such lesions may have been endoscopically resectable. By contrast, in our study, only 35% of the patients were diagnosed at stage 1 or earlier. In addition, late-stage rectal cancers were more common in our CAC cohort than in the general population. An epidemiological study in Taiwan demonstrated that CRC stage was significantly associated with survival rates (*P* < 0.001) (40). Early diagnosis is crucial because the mortality rate for colitis-associated rectal cancers was higher at 57.1% compared with sporadic rectal cancers at 26.8%.

Finally, we analyzed prognostic factors for poor outcomes. Our multivariable logistic regression results reveal that lesions with indistinct borders were the only statistically significant prognostic factor (adjusted OR = 11.5; 95% CI: 1.35–98.16; *P* = 0.03). This demonstrates the importance of careful morphological examinations. Other significant prognostic factors identified in our univariate analysis were rectal CAC, late-stage diagnosis, signet-ring cell carcinoma, unsuitability for endoscopic treatments, and receipt of chemotherapy.

This multicenter study, while providing in-depth clinical data unattainable in population-based studies, has limitations. The inherent heterogeneity stems from data collected over a span of years, encompassing shifts in treatment strategies, available medications, and surveillance policies. Comparing patients before and after 2011 (biological agent eligibility) was challenging because of the small pre-2011 sample. We addressed this by assessing adherence to surveillance and interval cancer rates based on the guidelines in effect at the time of diagnosis. In addition, the CAC diagnosis relied on IBD diagnosis chronology, without further pathological evidence such as immunohistochemistry staining for *P53* status. The self-reporting nature may lack traceability, potentially underestimating patient count. Referral center representation introduced selection bias toward severe population. Retrospective design led to unretrievable missing data and made it difficult to re-examine the pathology specimens. The UC-dominant cohort (92%) limited UC-CD CAC comparison. Further validation is necessary to generalize results to the broader IBD population.

In conclusion, this is the largest hospital-based study on the characteristics, management, and outcomes of CAC in Asia. On the basis of our findings, we recommend tight disease control and implementing strategies to reduce the occurrence of interval cancers. Individuals with CAC tended to receive diagnoses at younger ages and more frequently presented with signet-ring cell subtype, in contrast to sporadic CRC. Furthermore, rectal CAC was notably diagnosed at later stages, leading to poorer outcomes when compared with sporadic rectal cancers.

## CONFLICTS OF INTEREST

**Guarantor of the article:** Shu-Chen Wei, MD, PhD.

**Specific author contributions:** S.C.W. and T.Y.H.: conceived and designed the study. All authors contributed to data collection. H.Y.W.: analyzed the data. S.C.W., T.Y.H., and M.T.W.: led data interpretation, and all authors contributed. H.Y.W.: drafted the manuscript. S.C.W., T.Y.H., and M.T.W.: critically reviewed the manuscript. All authors approved the final version of the manuscript.

**Financial support:** This work was supported by a grant from National Taiwan University Hospital (MS507).

**Potential competing interests:** None to report.

**Data availability statement:** The data set used and analyzed during the current study is available from the corresponding author on reasonable request.

**Study Highlights**

**WHAT IS KNOWN**

✓ Patients with inflammatory bowel disease are at risk of colitis-associated colorectal cancer (CAC).
✓ The incidence and prevalence of inflammatory bowel disease are increasing in Asian countries.
✓ Medical and endoscopic advancements have led to decrease in CAC incidence in the West.

**WHAT IS NEW HERE**

✓ This is the largest Asian CAC cohort study.
✓ Signet-ring cell histology and interval cancers were not uncommon in CAC.
✓ Ill-defined lesions were 11.5 times more prone to adverse outcomes than well-defined lesions.
✓ Colitis-associated rectal cancers were diagnosed later and with worse outcomes than sporadic rectal cancers.

## Supplementary Material
