# Supplementary material for: Clinical Characteristics, Management, and Outcomes of Colitis-Associated Colorectal Cancer and the Comparison With Sporadic Colorectal Cancer in Taiwan
Source: Clin Transl Gastroenterol. 2024 Dec 5;16(2):e00798. doi: 10.14309/ctg.0000000000000798 (PMC11845191; doi:10.14309/ctg.0000000000000798)
Supplement: Supplementary file 1 [file ct9-16-e00798-s001.pdf]

SUPPLEMENTARY FIGURES

Supplementary Figure 1. Time to colitis-associated colorectal cancer development after onset of inflammatory bowel disease

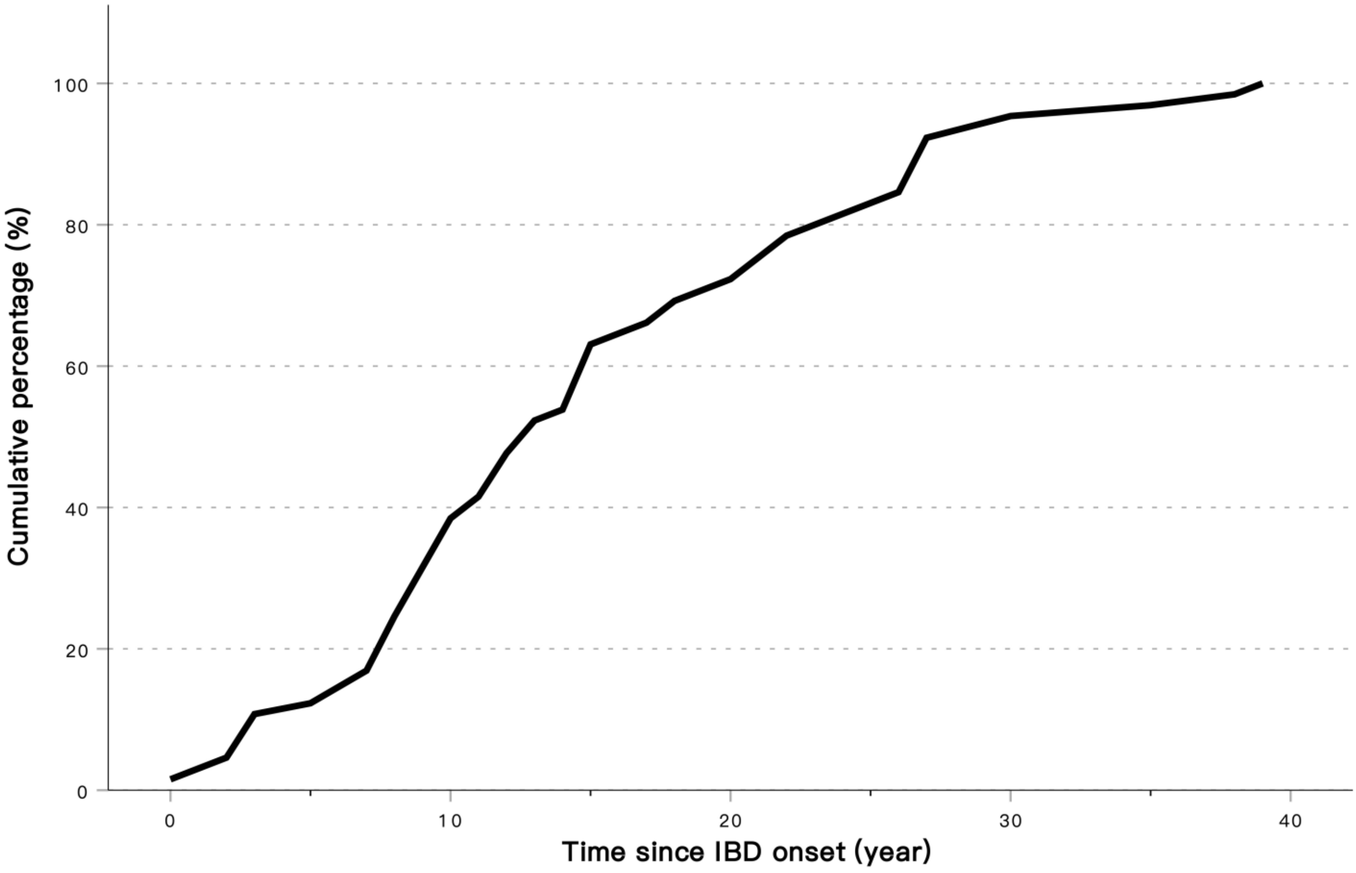

The risk of CAC increased parallelly with the duration of IBD. The median duration of IBD prior to CAC development was 13 years.

Supplementary Figure 2. Distinct morphology for colitis-associated colorectal cancers

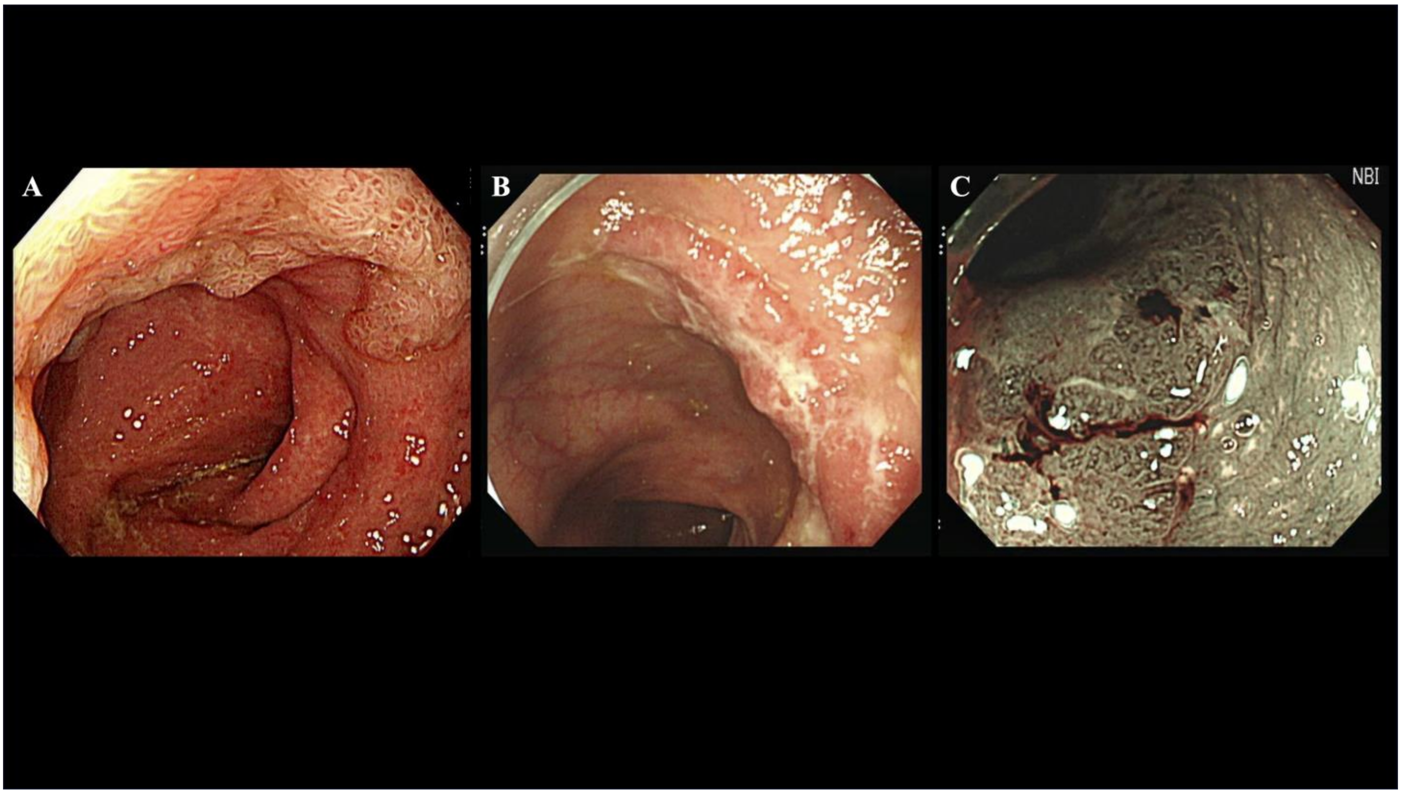

Figure 2A: Example of a pseudo-depressed nongranular type lateral spreading tumor, a non-polypoid lesion with distinct border under white light endoscopy. Figure 2B: Another non-polypoid lesion observed under white light endoscopy. Figure 2C: Improved border visibility of lesion using virtual chromoendoscopy with narrow band imaging.
